# Transposable elements at the center of the crossroads between embryogenesis, embryonic stem cells, reprogramming, and long non-coding RNAs

**DOI:** 10.1007/s11434-015-0905-x

**Published:** 2015-10-13

**Authors:** Andrew Paul Hutchins, Duanqing Pei

**Affiliations:** Key Laboratory of Regenerative Biology, South China Institute for Stem Cell Biology and Regenerative Medicine, Guangzhou Institutes of Biomedicine and Health, Chinese Academy of Sciences, Guangzhou, 510530 China; Guangdong Provincial Key Laboratory of Stem Cell and Regenerative Medicine, South China Institute for Stem Cell Biology and Regenerative Medicine, Guangzhou Institutes of Biomedicine and Health, Chinese Academy of Sciences, Guangzhou, 510530 China; Guangzhou Branch of the Supercomputing Center of Chinese Academy of Sciences, South China Institute for Stem Cell Biology and Regenerative Medicine, Guangzhou Institutes of Biomedicine and Health, Chinese Academy of Sciences, Guangzhou, 510530 China

**Keywords:** Transposable elements, Endogenous retroviruses, Embryonic stem cells, lncRNA, Reprogramming, Pluripotency

## Abstract

Transposable elements (TEs) are mobile genomic sequences of DNA capable of autonomous and non-autonomous duplication. TEs have been highly successful, and nearly half of the human genome now consists of various families of TEs. Originally thought to be non-functional, these elements have been co-opted by animal genomes to perform a variety of physiological functions ranging from TE-derived proteins acting directly in normal biological functions, to innovations in transcription factor logic and influence on epigenetic control of gene expression. During embryonic development, when the genome is epigenetically reprogrammed and DNA-demethylated, TEs are released from repression and show embryonic stage-specific expression, and in human and mouse embryos, intact TE-derived endogenous viral particles can even be detected. A similar process occurs during the reprogramming of somatic cells to pluripotent cells: When the somatic DNA is demethylated, TEs are released from repression. In embryonic stem cells (ESCs), where DNA is hypomethylated, an elaborate system of epigenetic control is employed to suppress TEs, a system that often overlaps with normal epigenetic control of ESC gene expression. Finally, many long non-coding RNAs (lncRNAs) involved in normal ESC function and those assisting or impairing reprogramming contain multiple TEs in their RNA. These TEs may act as regulatory units to recruit RNA-binding proteins and epigenetic modifiers. This review covers how TEs are interlinked with the epigenetic machinery and lncRNAs, and how these links influence each other to modulate aspects of ESCs, embryogenesis, and somatic cell reprogramming.

## TEs constitute a substantial proportion of the human genome

**Fig. 1:**
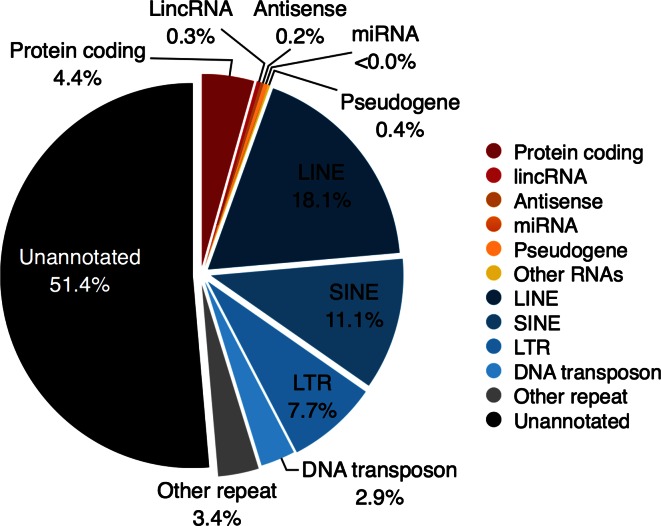
Estimated proportions of various selected genomic features within the human genome. Annotations were taken from GENCODE v23 with duplicate exons removed, and the UCSC genome browser “repeats and variations/rmsk” track for the human hg38 (GRCh38.p3) assembly. Base-pair numbers add up to greater than the sequenced genome size as some regions of DNA can overlap (e.g., lncRNAs and TEs, genes and TEs, antisense transcripts, and alternate splice sites). Consequently, the annotated features are somewhat overestimated and the unannotated genome underestimated

Mammalian genomes consist of a surprisingly high content of TEs. By counting the number of base pairs that appear within a specific genomic feature, such as a protein-coding gene, or repeat element, we can estimate that the human genome consists of approximately 51 % unannotated DNA, 4 % protein-coding genes and other regulatory RNAs, and nearly 40 % of the genome consists of TEs (Fig. 1). These numbers are in agreement with previous estimates [1–3] and reveal how successful TEs have been in propagating themselves in the human genome. Although TEs occupy nearly half of the genome, this is still an underestimate since computational techniques to detect TEs, such as RepeatMasker, have limited ability to identify ancient or divergent TEs. For example, the Xist lncRNA has several ancient TEs within its RNA that could only be identified using more sensitive methods [4]. Hence, as more sensitive techniques become available, and with a better understanding of the evolutionary history of genome sequences, the percent of identified TEs in the genome is likely to rise.

TEs can be classified into four major categories: DNA transposons and three classes of retrotransposon: long terminal repeat (LTR) containing endogenous retroviruses, long interspersed nuclear elements (LINEs), and short interspersed nuclear elements (SINEs) [5–7]. DNA transposons make up the smallest class of TEs (2.9 %; Fig. 1). The DNA transposons copy themselves by a “cut-and-paste” mechanism and rely on transposition during S phase for duplication. DNA transposons do not pass through an RNA intermediate, unlike the largest class of TEs, the retrotransposons (37 %; Fig. 1). LTR elements are endogenous retroviruses, and they are generally non-functional due to the accumulation of genomic mutations, although 16–18 ERVs are predicted to have a valid coding sequence for viral envelope proteins [8, 9], and there are many intact open-reading frames for viral capsids [10]. LINEs are the single largest category of TE (18 %). These TEs encode their own transposase, an enzyme required for TE duplication. Although most are non-functional due to mutation, it is estimated that at least 68 individual LINE-1 insertions are still active in human cells [11]. SINEs (11 %), conversely, do not encode their own transposase and instead rely on LINE encoded transposases to duplicate themselves. Consequently, they have sometimes been referred to as a “parasite’s parasite” [12].

Originally, TEs were thought to be non-functional, mainly parasitic elements, plaguing the genome, but there is a growing body of evidence that demonstrates roles for TEs in multiple biological processes. The most visible is the direct co-option (or exaptation) of endogenous retroviral genes for biological functions. For example, the syncytins (ERVWE1 in human, Syna/b in mouse) have been independently co-opted by evolution for a role in syncytium formation in the developing placenta [2]. The RAG1/2 enzymes critical for immunoglobulin V-D-J recombination in the immune system appear to be derived from transposases [13], along with several other examples of the co-option of viral genes for legitimate biological function [2]. Besides this direct use of the TE genes, evidence suggests that TEs themselves are involved in multiple aspects of early embryogenesis [14, 15], by forming regulatory elements to modulate epigenetic control [3], introduce alternate splice sites, provide evolutionary innovations in patterns of transcription-factor-binding sites [16, 17], influence genome evolution [5, 18], and may form functional regulatory domains in lncRNAs [19]. In this review we focus on the regulatory connections between TEs, epigenetics, and lncRNAs, and how these three facets are intimately linked with each other in the control of ESCs, reprogramming somatic cells to pluripotent stem cells, and early embryogenesis.

## TEs in embryonic development

TE expression has long been documented at various stages of embryonic development in the mouse [20, 21]. In the oocyte mRNA pool, a MaLR LTR may comprise up to 13 % of the total mRNA [22], and SINE elements may comprise a further 2 %–3 % [23]. Intact viral-like particles had long been observed under the electron microscope in mouse 2-cell embryos [24]. Still, it came as something of a shock to find viral-like structures in human embryos [25]. Although the human genome contains many intact open-reading frames for viral proteins [8–10], and a HERVK can be induced to from viral particles [26], no intact viral capsids had previously been observed in human embryos. These observations, coupled with genomic analysis, has focused research efforts on attempting to understand what possible roles these TEs play during early embryonic development, or whether they are just escaping epigenetic silencing when the embryonic genome is demethylated and reprogrammed. Genomic analysis of the RNA complement of developing embryos has been revealing. Expressed sequence tag (EST) data indicate the widespread expression of multiple classes of TEs at different embryonic stages [22, 27]. This has been elaborated recently by RNA-seq, which produces millions of short reads that can be mapped to the genome to more accurately locate TEs. This new technique has been applied to the analysis of single-cell RNA-seq data from human and mouse embryos and has revealed the highly specific expression of different classes of TE at different stages of human and mouse embryogenesis [28]. Even TEs within the same family show embryonic stage-specific expression. For example, for three LTR family members, LTR14B is restricted to the zygote, 2-cell and 4-cell stages, while LTR7B is mainly expressed at the 8-cell stage, and LTR7Y is expressed in the blastocyst [28].

It remains unclear the biological relevance of TE expression during early embryonic development, as few functional studies have been carried out. It is difficult to know in advance which specific TE to mutate, if the genome contains several tens or even hundreds of thousand of individual elements, each with different potential functions. Relatedly, only recently has genome-wide ultra-detailed maps of the temporal and tissue-specific expression of TEs become available [1, 28, 29]. For functionalizing TEs, one of the defining studies remains the observation that when MuERV-L transcripts are depleted from mouse oocytes, the developmental competence at the 4-cell stage is impaired [30]. This MuERV-L activity is time critical: The TE is expressed just 8–10 h after fertilization at the 2-cell stage, and although it is expressed up to the blastocyst stage, inhibition of MuERV-L after the critical 4-cell stage appears to have little effect on viability. Some clues to the requirements for TEs during early embryonic development can come from the experimental manipulation of epigenetic modulators and their effect on TE expression.

## TEs and epigenetic control in ESCs and the early embryo

DNA methylation is thought to be one of the major methods for somatic cells to suppress erroneous TE expression [31]. The early embryo undergoes dramatic epigenetic reorganization as the somatic genome is “reset,” and becomes ready for new rounds of differentiation and development in a process of near-global DNA demethylation. The widespread DNA demethylation in the early embryo consequently releases TEs from suppression and is a potentially hazardous event as the TEs can induce germline mutations. There is thus a conflict between the requirement for the erasure of epigenetic marks in the reprogrammed embryo and the resulting derepression of hazardous TEs [31]. Consequently, a widespread array of epigenetic suppression mechanisms, distinct from DNA methylation, is active in the early embryo and ESCs, and these mechanisms act to suppress TE activity. Several factors have been observed to bind to DNA and to recruit various epigenetic modifiers to specifically suppress TE expression [3, 14, 32].

### TRIM28 and epigenetic suppression of TEs

One of the best characterized suppressors of TEs is TRIM28 (KAP-1/TIF1b). TRIM28-knockout mice show embryonic lethality at E5.5 [33], and a maternal knockout of TRIM28 shows highly variable phenotypes, from early post-implantation lethality to a variety of growth abnormalities which result in no live births [34], although attribution of this effect to gene imprinting or TE suppression is unclear. TRIM28 is also required to maintain the suppression of TEs in ESCs, as the loss of TRIM28 leads to the deregulation of many TEs, and also developmentally regulated genes, even if relatively distal from TEs [35]. TRIM28 achieves this repression by recruiting the histone methyltransferase SETDB1 (ESET), heterochromatin protein 1 (HP1), and the deacetylase NuRD complex [36–38]. Together this complex achieves silencing of TEs, through methylation of histone H3K9 [35, 36], and via removal of the activatory histone acetylation epigenetic mark via the NuRD complex [36]. TRIM28 itself does not bind directly to DNA, instead it forms a docking platform for DNA-binding zinc finger proteins (ZFPs), which bind to TRIM28 through a KRAB (Kruppel-associated box) domain. TRIM28 has been associated with a series of ZFPs: ZFP809 [39, 40], YY1 (Yin Yang 1) [41], ZFP819 [42], and the essential pluripotency factor ZFP42 (Rex1) [43]. This widespread interaction with various ZFPs suggests some sort of code by which ZFPs suppress specific TEs. The ZFPs are the single largest family of putative transcription factors [44], and about 50 % of them contain a KRAB (TRIM28-interacting) domain [44]. The large number of ZFPs is thought to be a reflection of an evolutionary “arms race” between the TEs and the suppression machinery, an assertion supported by a correspondence between the number of TEs and the number of ZFPs in various vertebrate genomes, suggesting the co-evolution of TEs and suppressor complexes [45]. Recent work has highlighted this arms race between ZFPs and TEs, as shown by the rapid evolution of ZFP91 and ZFP93 to specifically suppress SVA SINE and L1 LINE elements, respectively [46]. ZFP91 and ZFP93 show modifications in their coding sequences in response to the emergence of these two TEs in primate genomes, 8–12 million years ago [46]. ZFPs appear to suppress TEs by binding directly to specific sequences inside the TEs themselves and recruiting epigenetic modifiers to suppress the TEs. Although not definitive for the hundreds of KRAB-containing ZFPs, among 18 KRAB-containing ZFPs analyzed by ChIP-seq, 16 showed enriched binding to various class-specific TEs [44]. Remarkably, from ZFP809 ChIP-seq data, the de novo consensus DNA-binding motif was a near perfect match to an endogenous retrovirus “PBS-pro” DNA sequence [40], implying that ZFPs specifically recognize TEs by binding to relevant sequences of DNA. It seems likely that the KRAB-containing ZFPs are a family of transcription factors tasked with specific suppression of TEs by recruiting TRIM28.

**Fig. 2:**
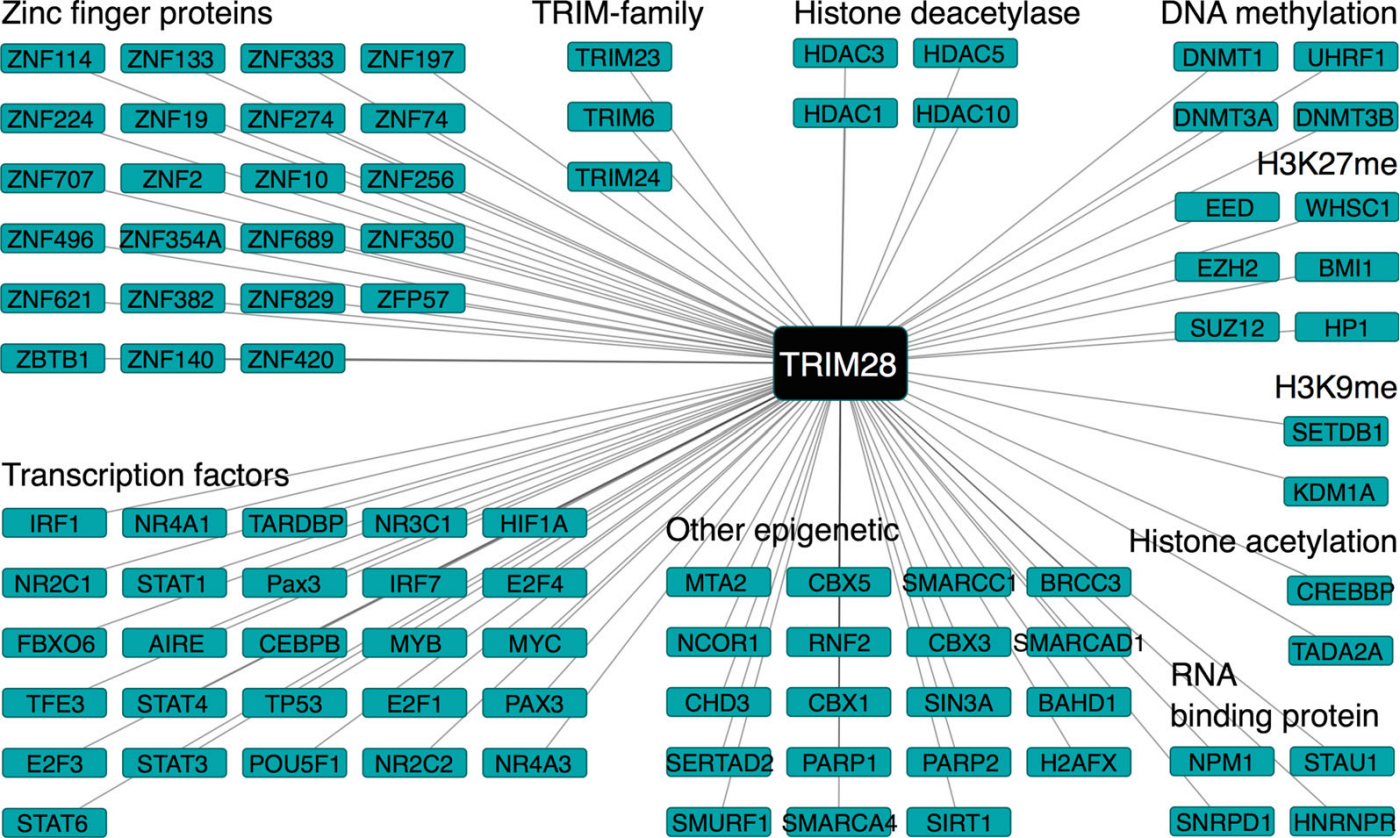
(Color online) TRIM28 is a binding platform for ZFPs and co-repressor/activator molecules protein–protein interaction data for TRIM28 (from BioGRID [48]). The Network shows selected first-degree interactions with TRIM28

**Fig. 3:**
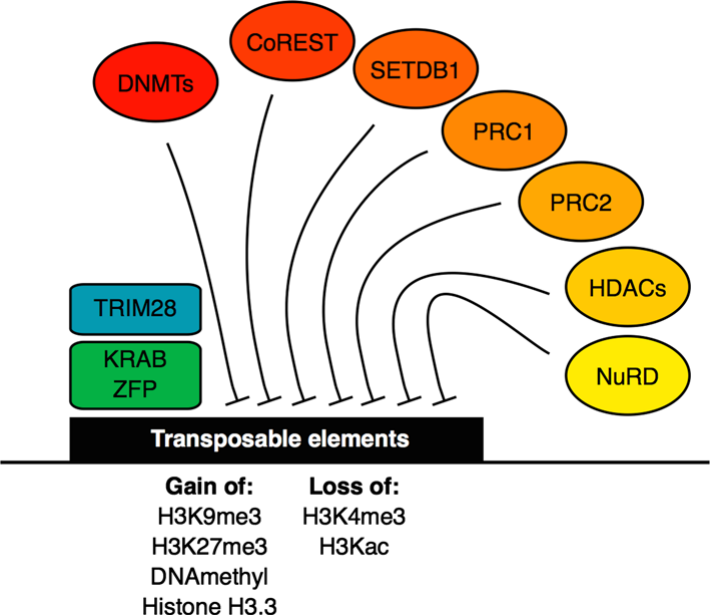
(Color online) Model of epigenetic suppression of transposable elements. KRAB-containing ZFPs are recruited to transposable elements, which then recruits the docking platform TRIM28 protein. This complex then recruits various co-repressor complexes, including (but not limited to) DNMTs, CoREST, SETDB1, PRC1, PRC2, HDACs, and NuRD. Other non-KRAB C2H2 ZFPs may also recruit co-repressors to TEs, particularly RYBP and YY1. These actions result in the gain of the repressive histone marks H3K27me3 and H3K9me3, the gain of variant histone H3.3 and DNA methylation to silence expression, along with the loss of the activatory H3K4me3 and Histone acetylation. Ultimately, many more epigenetic repressive mechanisms are likely to be involved in the suppression of TEs

TRIM28 acts as a docking platform for a wide array of co-repressor molecules ranging from histone methyltransferases, histone demethyltransferases histone deacetylases HDAC1, 2, 3, and the DNA methyltransferases DNMT3L [36, 47]. Protein–protein interaction data for TRIM28 [48] indicate that TRIM28 is also capable of interacting with many other potential regulatory proteins (Fig. 2). Among the TRIM28 interactors, many known functional interactions are present, particularly SETDB1 [36–38], KDM1A [49], and HDACs [50]. Additionally, TRIM28 can also interact with other epigenetic modifiers and even with transcription factors important in specifying cell type. For example, TRIM28 interacts with OCT4 (Pou5f1) [51], the master regulator of ESCs, and the early embryo [52]. TRIM28 also interacts with many ZFPs, possibly forming a regulatory code to identify specific TEs and suppress their expression [44]. Potentially, TRIM28 acts as more than just a docking platform for the suppression of TEs, but also integrates an elaborate regulatory network, targeted on the suppression of TEs (Fig. 3).

### Alternative histone modifications for the suppression of TEs

SETDB1 is not the only H3K9 methyltransferase involved in the suppression of TEs, SUV39H also methylates H3K9 to repress TEs, particularly LINEs [53], as can the H3K9 methyltransferases EHMT2 (G9A) and EHMT1 (GLP) [54], although their role in silencing IAPs is dispensable and SETDB1 is dominant [38]. Other histone methylations are also implicated in TE repression, H4K20me3 loss is seen on TRIM28 knockdown [35], and knockdown of the TRIM28/SETDB1 binding partner HNRNPK also results in loss of H4K20me3 at TEs [55], although H4K20me3 is not thought to be involved in the suppression of IAP TEs [38]. Loss of the histone H3K4 demethylase KDM1A (LSD1) also leads to up-regulation of repressed TEs by indirectly leading to inappropriate deposition of the activatory H3K4me3 and H3K27ac marks around TEs [49]. Intriguingly, co-immunoprecipitation of KDM1A identified a complex consisting of much of the CoREST complex (RCOR1, RCOR2, HDAC1, HDAC2, ZMYM2, PHF21A, HMG20B, and ZNF217) [50, 56], and additionally TRIM28 [49]. The presence of the CoREST complex and HDACs is interesting, suggesting they are deactylating TEs, which was supported as treatment of ESCs with HDAC inhibitors increases the expression of MERVL-family TEs [49].

### Alternative mechanisms for the suppression of TEs

TRIM28 is not the only regulator of TEs, and other alternative mechanisms are also involved. For example, APOBEC3B a cytidine deaminase RNA-editing enzyme is capable of suppressing the expression of LINE1 elements [57], elements that are active during the early phases of embryogenesis [58]. RYBP can also suppress TEs [59], possibly by recruiting the polycomb group repressors (PRC1, PRC2) via YY1 [60], and knocking out members of PRC1 or PRC2 results in the upregulation of MLV endogenous retroviruses [61]. Histone H3 can be replaced by the variant histone H3.3, the loss of which leads to the inappropriate derepression of IAP family LTRs in a process linked with H3K9me3 deposition [62] and so possibly TRIM28. When the chromatin-remodeling enzyme HELLS (LSH, a SNF2-like family member) is knocked out, it is embryonic lethal, and TEs show extensive DNA demethylation [63]. In addition to epigenetic control of TEs, RNA interference (RNAi) is also involved, as knocking down Dicer1 in early embryos leads to an up-regulation of MuERV-L at the 2-cell stage and IAPs at the 8-cell and blastocyst stages [64]. Similarly, Dicer1-knockout ESCs showed enhanced transcription from TEs, particularly IAP and LINE L1 elements [65]. RNAi and other small RNAs, such as piRNAs, have been proposed as “guardians of the genome” and play critical roles in maintaining the suppression of various families of TEs in embryonic and somatic tissues [66].

### Multiple epigenetic signatures and the control of TE suppression

It is clear that different types of TEs in ESCs harbor multiple distinct signatures of epigenetic modifications, i.e., specific combinations of the presence or absence of H3K4me3, H3K9me3, H4K20me3, and H3K36me3. Intriguingly, these histone-specific patterns are not only specific for TE families, but also show both cell-type-and family-type-specific signatures [67], indicating TE family-specific control of TE repression, even in somatic cells in which DNA methylation is thought to be the dominant suppressive mechanism [31]. Unfortunately, sequence reads mapping to multiple genomic sites (typically TEs) are often discarded early in ChIP-seq analysis pipelines, and consequently, the contribution of epigenetics and transcription factor binding to TE regulation remains woefully underestimated.

Ultimately, DNA methylation must be re-established in somatic tissues for the long-term stability of the genome. For example, DNMT1-null mice die around E9.5 and show 50- to 100-fold elevated levels of IAP RNA [68]. The KRAB-ZFPs are involved in the recruitment of the DNA methyltransferase enzymes [69] and may possibly help in reestablishing DNA methylation. It seems the global loss of DNA methylation in the early embryo has led to, or is at least concomitant with, the evolution of a system of elaborate epigenetic control in the early embryo (and in ESCs) [70], whose primary function is related to the careful repression of TEs and has a secondary role in controlling cellular differentiation and development. Ultimately, multiple overlapping mechanisms of epigenetic silencing are required to strictly control TE expression during early embryogenesis when the genome is being reprogrammed (Fig. 3).

## TEs in cell fate transitions and reprogramming somatic cells to pluripotent stem cells

Much in the same way that reprogramming of the embryonic genome releases the repression of TEs, something analogous happens as somatic cells are reprogrammed to induced pluripotent stem cells (iPSCs). LINE1 TEs are activated during reprogramming [71], along with many other families of TE in both mouse- and human-reprogramming experiments [72]. The functional role (if any) of this global derepression of TEs in pluripotent cell reprogramming remains unclear. Some clues can be gained by looking at the effect of knocking down epigenetic modification enzymes in reprogramming and ESCs. There are several studies showing that reducing the level of epigenetic factors can promote reprogramming, while knockdown of the same factors in ESCs causes the cells to become unstable and tend to differentiate. For example, knockdown of KDM1A (LSD1) enhances reprogramming [73], but its knockdown in ESCs promotes differentiation [74] via modulation of CoREST and HDAC activity [50] and grants ESCs an extra capability to differentiate toward an extraembryonic endoderm-like cell fate [49]. Similarly, inhibition of HDACs helps in reprogramming [75], while their inhibition in ESCs results in the up-regulation of TEs [49] and differentiation [76]. H3K9me3 itself is a major impediment in reprogramming somatic cells to pluripotency, leading to incompletely reprogrammed “pre-iPSCs.” These “pre-iPSCs” cells can be converted to fully reprogrammed iPSCs by using vitamin C [77], a process that is dramatically enhanced by knocking down H3K9 methyltransferases (and particularly SETDB1) [78]. This suggests that, cryptically, epigenetic remodeling and the derepression of TEs are linked and are both involved in cell fate transitions.

Intriguingly, the expression of a MuERV-L endogenous retrovirus marks out a very small population of ESCs that show similarity to the 2-cell stage of the developing embryo, and these cells have limited totipotent capability [79]. This process has been linked to the activity of the chromatin assembly factor 1 (CAF-1) complex, composed of Chaf1a (p150), Chaf1b (p60), and Rbbp4, which is involved in the correct deposition of histones H3 and H4 and assembly of heterochromatin. Loss of CAF-1 activity led to a substantial increase in the numbers of these 2C-like cells [80]. It is possible that the generation of these 2C-like cells is related to the extensive changes in heterochromatin seen in the transition from 2-cell embryos to cells of the blastocyst, a reorganization not observed in Chaf1a-mutant embryos [81].

The activation of TEs during reprogramming is of some concern as they have the potential to mutate the genome and so render patient-specific pluripotent cells oncogenic. Increased variability in the activity of TEs has been observed in different iPSC lines [72]. The release of TE suppression by deletion of the variant H3.3, which is associated with the repressive histone modification H3K9me3, leads to increased levels of chromosomal abnormalities [62]. An otherwise normal ESC line that had lost suppression of ERVs became incapable of germline transmission and resulted in chimeric mice with a characteristic “kinky tail” phenotype [82], that is reminiscent of phenotypes observed in mice with defects related to DNA methylation at LTRs. These results suggest that TE silencing is required for correct maintenance of pluripotency and chimera generation. It remains unclear whether the derepression of TEs is an absolutely essential event in somatic cell reprogramming or simply a side effect of global DNA demethylation. DNA demethylation itself is an essential requirement for the mesenchymal-to-epithelial transition (MET) [83], a critical event that occurs very early in the reprogramming process [84]. Knockouts of the three TET enzymes responsible for DNA demethylation leads to a block in the MET, and consequently reprogramming is also impaired [83]. Strategies to accelerate reprogramming [77, 85] are thus extremely valuable as they may help to minimize the window when TEs are active and capable of modifying the genome.

## TEs are lncRNAs, and lncRNAs are TEs

**Fig. 4:**
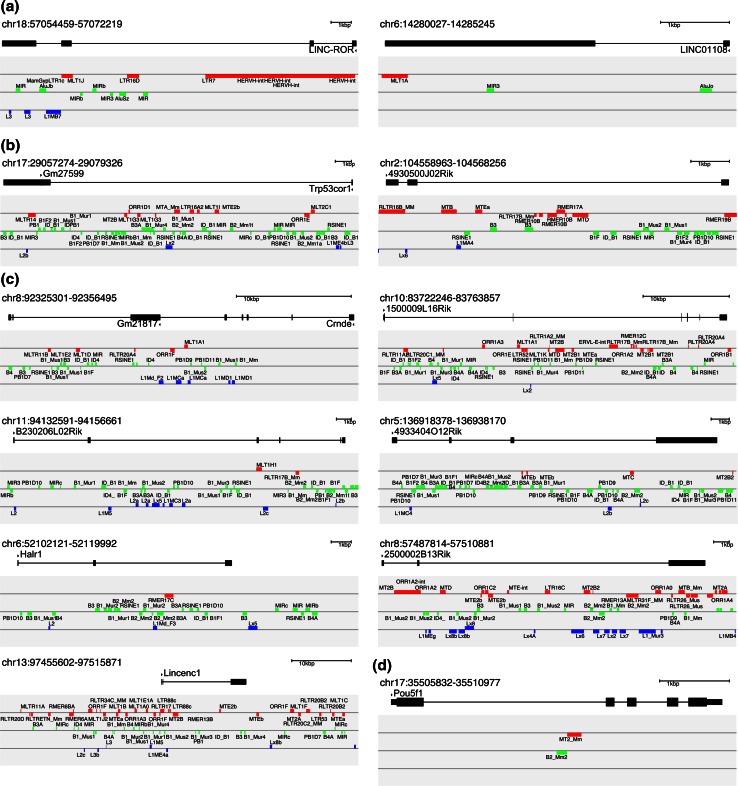
Genomic views of selected lncRNAs with demonstrated involvement in reprogramming or ESC maintenance reveal extensive presence of TEs. Genomic views indicate the gene; thick black parts indicate exons, which are connected with thin lines (introns). TEs are indicated across three lines in the gray panel, the top (red) indicates LTR endogenous retroviruses, the middle (green) indicates SINEs, and the bottom (blue) indicates LINEs. TEs above the light gray line are on the positive DNA strand and TEs below the light gray line are on the negative DNA strand. Some duplicate TE labels were removed for clarity. **a** LncRNAs involved in human iPSC reprogramming, LINC-ROR [89], and LINC01108 (linc-ES3) [91]. **b** Two mouse lncRNAs involved in reprogramming: Trp53cor1 (lincRNA-p21) [92] and 4930500J02Rik (Ladr83) [95]. **c** A selected series of lncRNAs involved in the maintenance of mouse ESCs [96]: Cnrde/Gm21817 (Linc1399), 1500009L16Rik (Linc1435), B230206L02Rik (Linc1448), 4933404O12Rik (Linc1543), Halr1 (Linc1547), 2500002B13Rik (Linc1577), and Lincenc1 (Linc1283). **d** The critical pluripotency gene Pou5f1 is shown for comparison. The protein-coding sequence is indicated with a thicker black line within the exons. Mouse genomic coordinates are mm10 and human are hg38 assemblies

Long noncoding RNAs (lncRNAs) are a class of gene that lack an obvious coding sequence, yet show many of the hallmarks of coding sequence genes, such as alternate splicing, and evolutionary conservation [86]. LncRNAs contain substantial components of TEs: 83 % of lncRNAs contain at least one TE, while of the total number of base pairs that comprise lncRNA sequences, 42 % is derived from TEs [87, 88]. Conversely, only 6 % of coding genes overlap with TEs [87, 88]. LncRNAs instead seem to match more closely to the genomic frequencies of TEs, albeit lncRNAs are depleted for particular classes of TE, such as L1 and enriched for others, such as MIR [87]. Several lncRNAs have been implicated as critical for ESC function and simultaneously are made of TEs. LINC-ROR, which modulates the efficiency of reprogramming [89], consists almost entirely of TEs (Fig. 4a), its transcription start site begins inside a HERVH element, and the LINC-ROR RNA contains further MLTIJ, L3, MIR, and other elements, while the introns contain multiple further MIR and Alu and other TEs [87]. LINC-ROR acts as a “sponge” to block the miRNA-mediated degradation of the critical pluripotency factors OCT4, SOX2, and NANOG [90]; of the five predicted miRNA-binding sites, four are within a TE including both of the experimentally confirmed miRNA-145-binding sites. LINC01108 (Linc-ES3) is required to maintain pluripotency [91] and contains two TEs (Fig. 4a). The mouse Trp53cor1 (lncRNA-p21), which is deleterious for reprogramming iPSCs [92], contains 7 TEs (Fig. 4b). LncRNAs can interact directly with the pluripotency machinery: Human L1TD1 is a lncRNA required to maintain pluripotency that is derived from the open-reading frame 1 of a LINE L1. It is capable of interacting with the pluripotency factor and RNA-binding protein LIN28A to modulate the levels of the pluripotent master regulator OCT4 [93], although L1TD1 is dispensable in mouse [94]. Genome-wide single-cell gene expression has revealed the widespread modulation of lncRNAs during reprogramming [95], and two lncRNAs in particular were identified as important in the reprogramming process: Gm16096 (Ladr49) and 4930500J02Rik (Ladr83), both of which contain TEs (Fig. 4b). This pattern extends for many other lncRNAs involved in the maintenance of the ESC state (Fig. 4c) [96]. However, as an example, the critical pluripotency gene Pou5f1 avoids any TEs inside its exons (Fig. 4d), although Nanog does contain SINE elements in its 3′UTR in both human and mouse.

An important caveat must be applied to research on TEs and lncRNAs. Experiments that use RNAi to knockdown entire classes of TE need to take some care as the RNAi may inadvertently also knockdown lncRNAs carrying the same TE that are essential for the maintenance of the pluripotent state. For example, HERVH-containing RNAs are specifically expressed in hESCs [97], are required for pluripotency [98], and, within the DNA, provide transcription-factor-binding sites for the naïve-specific LBP9 (also called Tfcp2l1) transcription factor [99]. Disruption of either LBP9, HERVH, or even HERVH-derived transcripts (novel RNAs derived from HERVH transcripts, some labeled as lncRNAs) led to the loss of pluripotency [99], yet LINC-ROR contains parts of a HERVH, and it is likely many other as yet undiscovered lncRNAs important for pluripotency also contain HERVH sequences. Consequently, knocking down the entire class of HERVHs in ESCs has the potential to also hit HERVH-containing lncRNAs that are essential for pluripotency.

It is a curious observation that many pluripotency-related lncRNAs begin their expression from a TE-derived promoter [88]. One possible explanation is that these TE-derived promoters may lead to the genesis of lncRNAs, starting from a TE-derived promoter and building up to a full lncRNA as new regulatory units and exons are assembled into a functional lncRNA. It remains unclear whether the lncRNA comes first, and then TEs colonize the functional lncRNA or instead TEs come first and assemble into a functional lncRNA [88]. Overall, it is clear that TE-containing lncRNAs play critical roles in the maintenance of pluripotency and the generation of iPSCs.

As TEs are often embedded in lncRNAs, this leads to the consequence that the literature discussing the biological functions of lncRNAs can also be considered as addressing potential regulatory or biological functions of TEs. It remains unclear exactly where TE expression ends and lncRNAs begin, and it is possible that researchers mapping the expression of TEs, using RepeatMasker TE annotations, may inadvertently be mapping lncRNAs, and calling them TE expression, while similarly, researchers de novo assembling RNA-seq data into novel transcripts may be assembling units of TE expression and calling them lncRNAs [100]. In that study, of the 3692 unannotated assembled transcripts, only 44 had robust expression and a predicted protein domain, the rest were unannotated transcripts that were enriched for various classes of TE [100]. The relationship between lncRNAs and TEs will need to be clearly defined as more RNA-seq datasets and better genome annotations become available [86].

### LncRNAs and regulation of chromatin modifiers

It is intriguing that lncRNAs physically interact with chromatin modifiers, particularly the H3K27 and H3K9 methyltransferases [96]. Considering these are the same enzymes responsible for suppressing TE expression [53, 54, 61], it is possible that the TEs role in modulating chromatin silencing for their own benefit has been co-opted by lncRNAs and used to control normal developmental programs. Indeed, lncRNA-p21 interacts with the H3K9 methyltransferase SETDB1 and the DNA methyltransferase DNMT1 during reprogramming to suppress pluripotency-related genes [92]. The binding of lincRNA-p21 to SETDB1 is mediated by HNRNPK, in a complex that can also specifically suppress TEs in ESCs [55]. That lncRNAs can recruit epigenetic modifiers is not unique to lncRNA-p21, and HOTAIR can recruit the PRC2 complex to methylate H3K27 and suppress gene expression at the HOX locus [101]. HERVH RNA in human ESCs can form scaffolds for the recruitment of co-activator complexes, particularly CBP, p300, MED6, MED12, OCT4, and CDK8, and HERVH expression is required both to establish the pluripotent state and to maintain it [98, 99]. The dynamic changes in lncRNAs observed during reprogramming [95] may also be related to the widespread epigenetic derepression of TEs during reprogramming [72]. For example, when KDM1A, an important enzyme for TE suppression, is knocked down in human somatic cells, they show enhanced reprogramming capability [73]. Similarly, a KDM1A inhibitor is included in the cocktail of molecules that can reprogram mouse somatic cells to pluripotent cells [102]. This suggests epigenetic control of reprogramming is also interconnected with mechanisms of TE suppression, although it remains unclear whether this beneficial effect on reprogramming is related to the derepression of TEs, or some other epigenetic process carried out by KDM1A during reprogramming.

### TEs as regulatory modules in lncRNAs

Although TEs are relatively rare in protein-coding genes [87, 88], they do show some bias toward the untranslated terminal region (UTR) of the mRNA [1]. This hints at a link to RNA-binding proteins and other levels of mRNA regulation. It is already known that the double-stranded RNA-binding protein Staufen (Stau1) can bind to an Alu–Alu stem loop to induce RNA degradation [103]. Relatedly, a systematic analysis of lncRNAs predicted TE-containing lncRNAs are more stable than non-TE-containing lncRNAs [88], suggesting a general role for TEs in modulating lncRNA degradation. Alu elements have also been demonstrated as critical for APTR-mediated suppression of the CDKN1A promoter by recruiting polycomb proteins to suppress its expression [104]. For other TE classes, a SINE element in the neuron-specific Uchl1 mRNA is required for posttranscriptional up-regulation [105]. It is not inconceivable that other TEs in protein coding or lncRNAs could serve as targets for RNA-binding-mediated regulation. HERVH RNA in hESCs is predicted to form a common domain that may potentially bind to other proteins, particularly pluripotent transcription factors [99]. It is possible to imagine that TEs residing inside lncRNAs act as regulatory elements for RNA-binding proteins, much in the same way that TEs in the genome harbor transcription-factor-binding sites [17]. The idea that TEs can form regulatory “domains” in lncRNAs has been expounded in the Repeat Insertion Domains of LncRNAs (RIDL) hypothesis [19], which posits that TEs form the regulatory modules inside lncRNAs, in an analogous fashion to structural domains in proteins. These domains can be swapped and exchanged between lncRNAs to innovate new biological functions [19]. This attractive hypothesis awaits validation, although many encouraging observations have been made. For example, a meta-analysis of RNA-binding protein CLIP-seq data uncovered extensive targeting of RNA-binding proteins to TEs [106]. Intriguingly, TEs can also act the other way around, as functional RNA-binding proteins themselves. For example, ESCs specifically express endogenous retrovirus HERVK [107], and a HERVK *Rec* protein can bind to the mRNAs of a wide array of signaling receptors (FGFR1, FGFR3, FGFR13, GDF3, and FZD7), chromatin modifiers (DNMT1, CHD4), and other RNA-binding proteins (LIN28A) important for pluripotency [25].

## The expanding links between TEs, lncRNAs, epigenetics, and embryogenesis

The links between lncRNAs, epigenetics, and embryogenesis are likely to grow in the future. The embryo, with its capability for germline transmission, is the site of vigorous competition between TEs and the host epigenetic suppression machinery as TEs vie to propagate their own DNA through the germ line. This vigorous competition has led to the co-option of TEs (perhaps as lncRNAs) and also the co-option of TE-mediated modulation of epigenetic regulation in normal developmental processes. Although an overarching model linking TEs, lncRNAs, and epigenetics remains lacking, and new data must be collected, it seems likely that the deep interconnection of TEs, lncRNAs, and embryogenesis will take yet more unexpected turns as surprising new observations emerge. As this review was going to press, an important study was published that systematically interrogated the factors in ESCs that are required for silencing of ERVs [108]. Using a genome-wide knockdown screen Yang and colleagues discovered hitherto unknown factors critical for the silencing of ERVs, particularly histone chaperones, alternate chromatin modifiers and intriguingly the sumoylation system. This study provides an excellent resource for the further study of TEs and provides many novel candidate mechanisms involved in the suppression of TEs.
